# Development and Validation of a Reversed-Phase Liquid Chromatography Method for the Simultaneous Determination of Indole-3-Acetic Acid, Indole-3-Pyruvic Acid, and Abscisic Acid in Barley (*Hordeum vulgare* L.)

**DOI:** 10.1155/2012/103575

**Published:** 2012-04-03

**Authors:** Ilva Nakurte, Anete Keisa, Nils Rostoks

**Affiliations:** Faculty of Biology, University of Latvia, 4 Kronvalda Boulevard, 1586 Riga, Latvia

## Abstract

A simple, sensitive, precise, and specific reverse HPLC method was developed and validated for the determination of plant hormones in barley (*Hordeum vulgare* L.). The method includes extraction in aqueous organic solvent followed by solid-phase extraction, sample evaporation, and reversed-phase HPLC analysis in a general purpose UV-visible (abscisic acid (ABA)) and fluorescence detection (indole-3-acetic acid (IAA) and indole-3-pyruvic acid (IPA)), high-performance liquid chromatography system. The separation was carried out on Zorbax Eclipse XDB C8 column (150  ×  4.6 mm I.D) with a mobile phase composed of methanol and 1% acetic acid (60 : 40 v/v) in isocratic mode at a flow rate of 1 ml min^−1^. The detection was monitored at 270 nm (ABA) and at 282 nm (Ex) and 360 nm (Em) (IAA, IPA). The developed method was validated in terms of accuracy, precision, linearity, limit of detection, limit of quantification, and robustness. The determined validation parameters are in the commonly acceptable ranges for that kind of analysis.

## 1. Introduction

Auxins are a group of ubiquitous and vital plant phytohormones regulating plant growth and development [1, 2] and participating in plant response to biotic [3, 4] and abiotic stimuli [5].Although there are several naturally occurring compounds comprising auxin activity, indole-3-acetic acid (IAA) is the most abundant auxin [6]. Only a minor part of IAA in plant resides in free form, the majority being conjugated to amino acids or sugars [7]. Composition of auxin pool can yield physiologically relevant information on actual state of auxin biosynthesis and signaling in plants [8]. Physiological and molecular mode of action of auxin has mainly been studied in dicot model species *Arabidopsis thaliana *[9]. Monocots exhibit slightly different auxin response [10]; therefore, information gained from auxin studies in *A. thaliana* need to be confirmed in agriculturally relevant monocot species, such as barley, wheat, or rice. Although molecular biology approach using gene expression studies or induced mutagenesis have provided a certain insight into auxin signaling and biosynthesis, application of high-performance liquid chromatography (HPLC) and mass spectrometry (MS) methods for qualitative detection and quantification of various forms of auxin would significantly contribute to auxin studies by supplying direct evidence of an actual state of auxin pool *in planta*. In addition, HPLC and MS allow for detection of multiple compounds in the same assay, if compounds of similar chemical properties are analyzed. Several plant phytohormones have similar chemical properties to IAA suggesting that method allowing for detection of IAA would also be applicable to other phytohormones. Abscisic acid is a plant phytohormone involved in regulation of plant stress responses as well as participating in developmental processes [11]. ABA is one of the main components ensuring stomatal closure in response to unfavorable environmental conditions and pathogen attack [12]. Interestingly, auxin is able to repress stomatal closure through its antagonistic effect on ABA [13]. Phytohormones often exhibit contrasting effects on different physiological functions; for example, ABA regulates transpiration by causing stomatal closure, while auxin typically is a positive regulator of stomatal opening at low concentrations, but an inhibitor at high concentrations [14]. Therefore, analyzing both IAA and ABA in the same sample would offer excellent opportunity to monitor crosstalk between the two pathways regulating stomatal movements in response to various stressors. Recently, reversed-phase HPLC has become the most preferable method for the separation and determination of plant hormones, because of its simplicity, rapidity, and reliability [15–17]. The difficulty of auxin analyses arises from their presence in minute quantities among the bulk of other substances in plant extracts [18]. Therefore, the determination of some plant hormones in biological material requires extensive purification prior to final quantification step [19]. Liquid-liquid extraction is one of the simplest methods for sample purification [17], but the biggest disadvantage of this method is the loss of compound of interest. Solid-phase extraction (SPE) is a well-established method routinely used for cleanup and preconcentration step of compounds. SPE using C18 bound silica has been frequently used as an efficient method for purification of plant hormones on the basis of reversed-phase interactions [20, 21]. In this paper, we evaluated the method for IAA, IPA, and ABA extraction, purification and quantification in the monocotyledonous plant barley. Once the HPLC method development was completed, the method was validated. 

## 2. Experimental

### 2.1. Materials and Reagents

**Figure 1:**
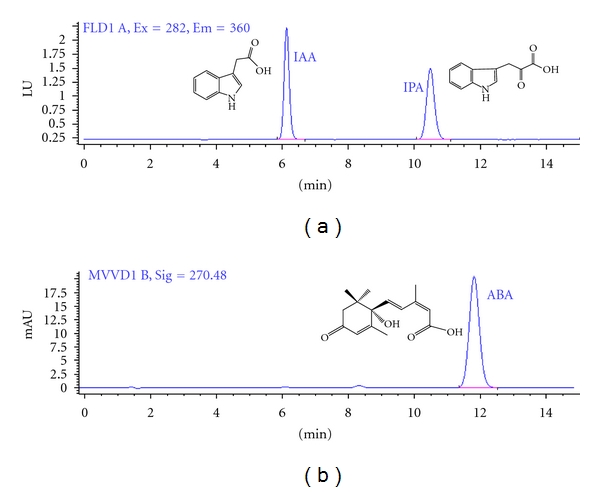
The chromatogram of isocratic separation of indole-3-acetic acid, indole-3-pyruvic acid, and abscisic acid in test mixture and their structures.

Acetonitrile, methanol (both gradient grade), and acetic acid (≥99%) were supplied by Sigma-Aldrich (St. Louis, USA). The water used in this work was purified with a Milli-Q water purification system from Millipore (Bedford, USA). Standards of indole-3-acetic acid (>99%), indole-3-pyruvic acid (99%), and abscisic acid (99%) were purchased from Sigma-Aldrich (St. Louis, USA). The molecular structures of compounds are shown in Figure 1. Stock solutions of the individual standards at a concentration of 0.1 mg mL^−1^ were prepared by dissolving the compounds in acetonitrile and were stored at 4°C. Working solutions of mixtures of all the standards were prepared immediately before analyses by diluting the stock solution with mobile phase, to attain the required concentrations for calibration measurements.

### 2.2. Instrumentation

Chromatographic analysis was performed on a modular HPLC system, Agilent 1100 series consisting of quaternary pump, autosampler, column thermostat, UV and fluorescence detectors (Agilent Technologies, Germany). HPLC separations were achieved by using a reverse-phase Zorbax Eclipse XDB-C8 (Agilent Technologies, Germany) column 4.6 × 150 mm, 5 *μ*m. Colum temperature was controlled at 30°C. Mobile phase was composed of methanol and 1% acetic acid (60 : 40 v v^−1^) in isocratic mode at a flow rate of 1 mL min^−1^. The detection was monitored at 270 nm (ABA) and at 282 nm (Ex) 360 nm (Em) (IAA, IPA). Injection volume was 50 *μ*L. Results were evaluated by a ChemStation Plus (*Agilent, Germany*).

### 2.3. Plant Material

Seeds of *Hordeum vulgare* cultivar “Parkland” were surface sterilized in 2.5% bleach solution and incubated at 4°C for 3 days to synchronize germination. For leaf tissue samples, seedlings were grown in soil at 22°C under long-day (16 h day, 8 h night), medium light (ca. 150 *μ*mol m^−2^ s^−1^) conditions. Leaf samples were taken from 14 days old plants.

### 2.4. Sample Preparation

Two different methods of extraction and purification for auxin were adapted from [17, 19] and later compared. Briefly, the plant material was ground in liquid nitrogen. Ground samples were weighted and extracted with 100% methanol (2.5 mL per gram of fresh weight (g.f.w.)). The extract was cleared by centrifugation (4,000 g × 10 min) at room temperature. The resulting supernatant was transferred to a new tube and evaporated until the volume decreased to less than one-tenth of the initial.

### 2.5. Liquid-Liquid Extraction

According to [17], one volume of pure water was added to residue. The pH was adjusted higher than 9 with 1 M KOH to keep IAA and IPA ionized and then partitioned against 100% ethyl acetate. The aqueous and organic phases were separated by centrifugation (16,000 g × 5 min), and the lower aqueous phase was transferred to a new tube. The pH of the solution was lowered below 3 with concentrated acetic acid to conserve IAA and IPA in protonated forms. The acidic sample was partitioned against 100% ethyl acetate and cleared by centrifugation. The upper organic phase was recovered and completely evaporated and then dissolved in 300 *μ*L of the mobile phase.

### 2.6. Extraction Using C18 SPE

The evaporated residue was dissolved in a 1% acetic acid solution (2.5 mL/g.f.w). Solution was filtered with 0.20 *μ*m filters (Nonpyrogenic Sterile-R, Sarstedt), to remove particulate and other suspended solid matter. The filtered samples were immediately preconcentrated by SPE using AccuBOND II ODS-C18 200 mg 3 mL SPE (Agilent). C18 SPE columns were pretreated with 2.5 mL of methanol followed by 2.5 mL of 1 M acetic acid. Then, samples (approximately 2.5 mL) were loaded on the cartridge. The column was washed with 2.5 mL of 1 M acetic acid. Methanol (2.5 mL) was used to elute the analytes from the extraction column. The extract was evaporated till dryness and redissolved in 300 *μ*L of the mobile phase.

### 2.7. Method Validation

The method was validated in terms of accuracy, precision, linearity, limit of detection, limit of quantification, and robustness.

#### 2.7.1. Linearity and Range

The linearity of measurement was evaluated by analyzing different concentrations of the standard solutions of the IAA, IPA, and ABA. Calibration standards were prepared by diluting the stock solutions to obtain the concentrations indicated in Table 2. The detection and quantification limits were calculated as the peak height at a signal-to-noise (S/N) ratio of 3 and 10, respectively. All solutions were prepared in a mixture of mobile phase.

#### 2.7.2. Accuracy

Recovery studies were performed with barley leave samples spiked at 50% (50 ng mL^−1^), 100% (100 ng mL^−1^) and 150% (150 ng mL^−1^), levels with all stock solutions prepared.

#### 2.7.3. Method Robustness

To study robustness of the method, conditions of HPLC method, such as percentage of methanol in mobile phase (from 30 to 60%), pH of buffer (from pH 2.0 to pH 7.0), and column temperature (from 25°C to 50°C), were changed. The data are presented as the relation between the changed parameter versus the l*g *of capacity factor *k*. Finally, stability of sample and standard solutions at room temperature (25°C) were detected during 24 hours.

## 3. Results and Discussion

Different HPLC methods have been described for separation and detection of IAA, IPA, and ABA [16, 17]. We tried to develop a simple and specific method for simultaneous extraction and determination of IAA, IPA, and ABA. Compounds were analyzed on a reversed phase HPLC column under isocratic program in the presence of 1% acetic acid and methanol as described in experimental section. We used both fluorescence and UV monitoring, and consistently with other studies [17], the fluorescence signal was 10 times stronger than UV absorption for IAA and IPA with the same amount of material (data not shown). Final elution was traced with fluorescence detector for IAA and IPA detection. ABA was not detectable using fluorescence detector; therefore, UV detection was used for this compound. Under the described HPLC conditions, the IAA, IPA, and ABA were fully separated in 15 min with symmetrical peaks at 6.2 min, 10.6 min, and 11.9 min, respectively (Figure 1). 

The purification and separation of auxins and ABA from other plant hormones is a difficult task. Several different methods have been described, using either extraction with organic solvents [17] or purification with different solid phase cartridges [16]. We tried two different approaches—simple liquid-liquid extraction (Method 1) with serial partitioning and evaporation as well as extraction with organic solvent and purification using SPE cartridges (Method 2). We spiked our barley samples with known amount (50 ng mL^−1^, 100 ng mL^−1^, and 150 ng mL^−1^) of IAA, IPA, and ABA standard solutions and then conducted the HPLC analysis. The contents were determined from the respective chromatograms. The concentration of the compound was determined using external standards. The recovery % was calculated by using the following formula:

(1) $$\%=\frac{\left(b-a\right)}{c\;}*100,$$

where

- The amount of compound before the addition of a standard;
- The amount of compound after the addition of a standard;
- The amount of standard compound added.

The obtained results were satisfactory and are presented in Table 1. These data were used as accuracy data for validation as well.

While both methods can be used for separation of IAA, IPA, and ABA, the loss of determined compounds with SPE method (Method 2) ranged from 1.3 to 2.5%, but the loss with Method 1 was significantly higher ranging from 30.8 to 40.1%. As there may be a number of explanations for the difference between the two methods, several different types of quantification have been used. In HPLC-based quantification without MS, radio-labelled internal standards are generally included [16, 22]; for example, in [17], all the values were corrected against the signal of internal standard. Our evaluated method using external standards showed high recovery. Once the HPLC method development was over, the method was validated in terms of accuracy, precision, linearity, limit of detection, limit of quantification, and robustness. The data are represented in Table 2.

The linearity was measured by analyzing six different concentrations of the standard solutions of the IAA, IPA, and ABA. Calibration curve was constructed by plotting average peak area against concentration, and regression equation was computed. The assay provided a linear response across a wide range of concentrations. LOD and LOQ were determined from calibration curves, and it was concluded that the developed method is sensitive. A mixture of standard solutions was injected six times, and corresponding peak areas were recorded. The % of relative standard deviation (RSD) was found to be less than 1%. The high percentage in recovery studies described before indicates the accuracy of the method. 

**Figure 2:**
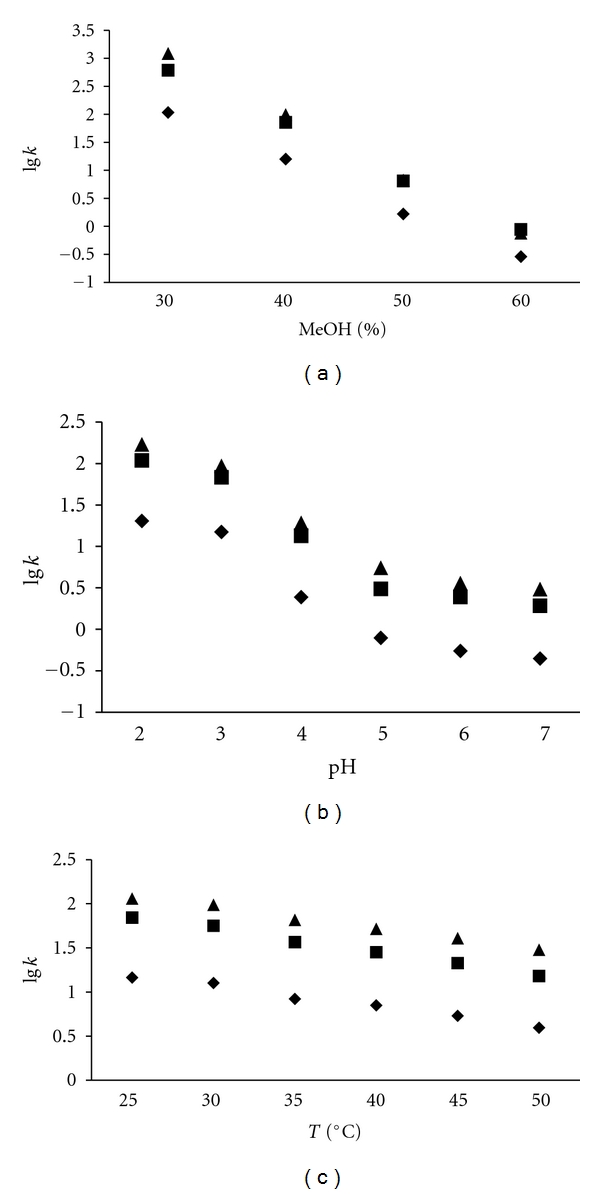
The influence of methanol % v/v concentration (a), mobile phase pH (b), and temperature (c) on the retention factors of IAA (♦), IPA (■), and ABA (▲).

In order to assess robustness of the developed method HPLC, several parameters, such as percentage of methanol in mobile phase, pH of buffer, and column temperature, were changed. The data are presented as the relation between the changed parameter versus the l*g *of capacity factor *k *(Figure 2). 

All analyzed hormones followed the reversed-phase behavior, while being separated. In spite of the introduced changes, no additional peaks were found, although there were shifts in retention times or small changes in peak shapes. It was evident that the presence of some excess amount of the organic modifier close to the adsorbent surface decreased the hormone interaction and also retention. Several plant hormones have acidic and/or basic properties and IAA, IPA, and ABA ionization state can be controlled by the pH of mobile phase and that confirms findings of other authors [15, 19, 23]. Changes in column temperature did not exhibit large influence on the retention factors of the compounds investigated, affecting predominantly asymmetry and column efficiency. 

The developed method allowed quantification of IAA, IPA, and ABA in different barley tissue samples and also in other plant species, such as model organism thale cress (*Arabidopsis thaliana* (L.) Heynh.) (data not shown), providing a useful tool for simultaneous study of IAA, IPA, and ABA. 

Analysis of the phytohormones in shoots of barley (cultivar “Parkland”) showed content of 14.03 ± 1.84 ng g^−1^ f.w. IAA, while roots contained 2.42 ± 0.78 ng g^−1^ f.w. IAA. The concentration of ABA was below detection threshold both in shoots and roots, while IPA was not found in both.

## 4. Conclusion 

Based upon the results of the current study, we conclude that simultaneous extraction, separation, and quantitative determination of IAA, IPA, and ABA from barley are possible using SPE method. The determined validation parameters for developed method are in the commonly acceptable ranges for that kind of analysis. The good percentage recovery indicates the accuracy of the method, while the concentrations of hormones can be accurately and precisely quantified with a very low limit of detection. Hence, the proposed method is simple, accurate, and rapid and can be employed to routinely study auxin and ABA concentration in barley (*Hordeum vulgare* L.) and possibly in related Triticeae species, such as wheat or rye. The developed method will serve as a convenient tool for studying auxin effects on plant development and interactions with microorganisms.

## Figures and Tables

**Table 1:** Recovery studies of IAA, IPA, and ABA in spiked barley samples.

%	Average (*n* = 3) ± SD		Average (*n* = 3) ± SD		Average (*n* = 3) ± SD		Average (*n* = 9) ± SD	
	50 ng mL^−1^ = 50%		100 ng mL^−1^ = 100%		150 ng mL^−1^ = 150%			
	Method 1	Method 2	Method 1	Method 2	Method 1	Method 2	Method 1	Method 2
IAA	56.4 ± 3.5	96.8 ± 2.9	59.8 ± 3.3	97.1 ± 1.8	63.5 ± 3.6	99.6 ± 2.2	59.9 ± 3.6	97.5 ± 1.5
IPA	65.5 ± 2.4	97.9 ± 2.2	60.2 ± 3.6	96.8 ± 2.9	64.4 ± 2.9	99.4 ± 2.0	63.4 ± 2.8	98.7 ± 1.3
ABA	68.4 ± 3.4	95.1 ± 3.1	72.1 ± 4.1	99.3 ± 2.2	67.2 ± 3.3	100.1 ± 2.8	69.2 ± 2.6	98.2 ± 2.7

**Table 2:** Validation parameters.

Parameter	Results		
	IAA	IPA	ABA
Linearity range, ng mL^−1^	1–10000	5–10000	5–10000
Correlation coefficient	0.9997	0.9997	0.9998
Equation	*y* = 0.0236*x* − 0.0831	*y* = 0.0211*x* − 0.5677	*y* = 0.4456*x* − 14.576
LOD, ng mL^−1^	1.82	5.16	5.85
LOQ, ng mL^−1^	5.51	15.64	17.73
Recovery, %	97.5 ± 1.5	98.7 ± 1.3	98.2 ± 2.7
Stability	Stable over 24 h at room temperature, RSD less than 2.0%		
